# Cardioprotective role of royal jelly in the prevention of celecoxib-mediated cardiotoxicity in adult male albino rats

**DOI:** 10.1186/s13019-024-02593-2

**Published:** 2024-03-18

**Authors:** Naglaa Z. H. Eleiwa, Hesham A. M. I. Khalifa, Heba A. Nazim

**Affiliations:** Department of Pharmacology, Faculty of Vet. Med, Zagazig University, Zagazig, 43511 Egypt

**Keywords:** Cardiac, Celecoxib, COX-2 inhibitor, Royal Jelly

## Abstract

**Background:**

Celecoxib, a cyclooxygenase-2 selective inhibitor non-steroidal anti-inflammatory drugs, is used for the management of short- and long-term pain as well as in other inflammatory conditions. Unfortunately, its chronic use is highly associated with serious abnormal cardiovascular events. The current study was designed to explore the effect of long-term administration of celecoxib on the cardiac tissues of male albino rats. The study also examined the alleged cardioprotective effect of royal jelly.

**Methods:**

Thirty, male albino rats were randomly divided into 3 equal groups; 10 each: (1) rats served as the control group and received no drug; (2) rats received celecoxib (50 mg/kg/day, orally), for 30 consecutive days; (3) rats received celecoxib (50 mg/kg/day, orally) plus royal jelly (300 mg/kg/day, orally) for 30 consecutive days. Sera were collected to assay cardiac enzymes and oxidant/antioxidant status. Rats were euthanatized and cardiac tissues were dissected for quantitative estimation of apoptotic genes (Bax) and anti-apoptotic gene (Bcl-2).

**Results:**

Long-term celecoxib administration caused cardiotoxicity in male albino rats as manifested by significant elevation of serum levels of creatine phosphokinase (CPK), creatine kinase-MB (CK-MB), and lactate dehydrogenase (LDH), with ameliorative effects of royal jelly against celecoxib-induced cardiotoxicity as manifested by significantly decrease in serum CPK, CK-MB, and LDH levels. It also showed a significant decrease in the oxidative stress indicator malondialdehyde (MDA) levels and the bax gene. Additionally, it demonstrated significant increases in the bcl-2 gene and superoxide dismutase (SOD) levels, which contribute to its therapeutic effects against celecoxib-induced cardiotoxicity.

**Conclusion:**

Long-term celecoxib administration caused cardiotoxicity in male albino rats with protective effect of royal jelly being given together. It could be concluded that royal jelly may prove a useful adjunct in patients being prescribed celecoxib.

**Trial registration:**

Not applicable.

## Background

Celecoxib, a cyclooxygenase-2 (COX-2) selective inhibitor, is a prospective alternative for the treatment of osteoarthritis, rheumatoid arthritis, and ankylosing spondylitis [1]. Following its introduction to the United States (US) market in December 1998 after FDA approval, celecoxib swiftly became one of the most often prescribed medications for the treatment of various forms of arthritis and the management of acute or chronic pain due to its favorable gastrointestinal toxicity profile [2].

The adverse effects of celecoxib, still require further in-depth study. The results of studies about the effect of celecoxib on the heart varied considerably. While one study proved the harmful effect of celecoxib on the heart in the form of an increased risk of myocardial infarction [3], another study confirmed that there was no noticeable effect on the heart [4]. Since 2004, when two COX-2-selective inhibitors, rofecoxib, and valdecoxib, were removed from the market due to an elevated risk of cardiovascular events, including myocardial infarction, celecoxib, as well as other selective and nonselective Non-steroidal anti-inflammatory drugs (NSAIDs), have been under intensive examination [5]. Celecoxib is the only powerful, selective COX-2 inhibitor (among the “coxib” family) that is still commercially accessible [6]. As a result, celecoxib became a commercial prototype inhibitor for the development of COX-2 enzyme anti-inflammatory medicines [7].

Royal jelly (RJ) is a nutrient-rich source of bioactive compounds that are essential to many biological processes. About (60–70% w/w) water, (9–18% w/w) proteins, (7–18% w/w) carbohydrates, and (3–8% w/w) lipids make up the majority of RJ. Minor components also found in RJ include vitamins (A, B complex, C, and E), Minerals (Fe, Zn, Mg, Mn, Ca, Na, K, and Cu), Amino acids (lysine, cysteine, glycine, proline, valine, serine, cysteine, alanine, tyrosine, threonine, leucine, isoleucine, glutamine, phenylalanine, hydroxyproline, aspartic acid, and glutamic acid), hormones, enzymes, nucleotides, polyphenols, and minor heterocyclic compounds [8]. RJ has potential health benefits, such as antimicrobial, antioxidant, anti-inflammatory, anticancer, antihyperlipidemic, cardio-protective, and hepatorenal-protective properties [9]. Free radical scavenging, suppression of oxidative and nitrosative stress, and anti-apoptotic ability are all thought to contribute to the cardioprotective effect of royal jelly [10–12].

With special concern for the cardioprotective effect of royal jelly administration, the current study was designed to explore the effect of long-term administration of celecoxib on cardiac tissues in male albino rats. In addition, to explore the effect of royal jelly on oxidant/antioxidant status regarding malondialdehyde **(MDA)**, serum superoxide dismutase **(SOD)** and its effect on apoptotic genes **(Bax)** and anti-apoptotic gene **(Bcl-2)** expression.

## Methods

### Experimental animals

Thirty, apparently healthy, male albino rats weighing 200–230 g, randomly divided into three equal groups; each of 10 rats, were employed in this study. They were contained in clean cages with wire-bottomed galvanized metal walls. They were given a balanced diet and unlimited access to fresh tap water. Prior to commencement of the study, they had a 7-day acclimatization period.

### Study design and setting

This study was conducted in the Lab animal unit, Pharmacology department, Faculty of Veterinary Medicine, Zagazig University, Zagazig, Egypt, in the period from March 2023 to May 2023. Zagazig University Scientific Research and Publications Ethics Committee approved the study. All steps and procedures in the study were carried out following Zagazig University Institutional Animal Care and Use Committee regulations, with approval No. ZU-IACUC/2/F/ 522 /2023.

Group 1(control group): rats received no drug. Rats of this group served as the control group. Group 2 (celecoxib group): rats received celecoxib (Pfizer Co., Egypt) (50 mg/kg/day, orally) [13], for 30 consecutive days, then rats were sacrificed and samples were collected. Group 3 (celecoxib + royal jelly group): rats received celecoxib (50 mg/kg/day, orally) [13], and royal jelly (Pharco Co., Egypt) (300 mg/kg/day, orally) [9] for 30 consecutive days, then rats were sacrificed and samples were collected.

### Samples collection and preservation

#### Blood samples

Blood samples were collected using a 3 ml syringe directly from the ventricular puncture of rats into centrifuge tubes and left to clot for 15 min at room temperature then centrifuged at 3000 rpm for 10 min to allow serum separation, which was then aspirated into cryovials and stored at -20 °C for serum biochemical assays of cardiac enzymes and oxidant/antioxidant status.

#### Tissue specimens

Rats were euthanatized using sodium thiopental (200 mg/kg Bwt, I/P) for sampling. The cardiac tissues of each animal were dissected and cut into small pieces. About 30 mg of the rat cardiac tissue samples were immediately collected, transferred in liquid nitrogen, and kept at – 80 ◦C for total RNA extraction used for the determination of apoptotic and anti-apoptotic mRNA expression levels using RT-PCR [14].

### Biochemical measurements in sera

#### Measurement of serum cardiac biomarkers concentrations

Quantitative estimation of serum creatine phosphokinase concentration **(CPK)**, serum creatine kinase-myocardium bound concentration **(CK-MB)**, and serum Lactate dehydrogenase concentration **(LDH)** were carried out automatically by Roche/Hitachi Cobas® c systems and Cobas® 6000 analyzer.

#### Oxidant/antioxidant status

Quantitative estimation of serum malondialdehyde **(MDA)**, and serum superoxide dismutase **(SOD)** concentrations were carried out using commercially available kits supplied from Oxi Select™, USA [15].

### Apoptotic and anti-apoptotic genes expression

TOPreal™ qPCR 2X PreMIX (SYBR Green with low ROX) (Cat. # P725, Enzynomics, Korea) was used for quantitative estimation of apoptotic genes **(Bax)** and anti-apoptotic gene **(Bcl-2)** based on the manufacturer’s protocol.

Gene expressions were measured using the below formula and Ct (2-ΔΔCt) (fold change) [14].

$$\begin{array}{l}{\bf{\Delta \Delta Ct}}\, = \,\left( {{\bf{C}}{{\bf{t}}_{{\bf{target}}}} - {\bf{C}}{{\bf{t}}_{{\bf{reference}}}}} \right)\,{\bf{test}}\,{\bf{sample}} - \\\left( {{\bf{C}}{{\bf{t}}_{{\bf{target}}}} - {\bf{C}}{{\bf{t}}_{{\bf{reference}}}}} \right)\,{\bf{control}}\,{\bf{sample}}\end{array}$$

Finally, considering the primer efficiency value of ~ 2, the gene expression level was determined as 1-ΔΔCt.

### Statistical analysis

The obtained data were analyzed statistically using the Statistical Package for Social Sciences (SPSS) program (version 26.0; SPSS Inc, IL, USA) for Microsoft Windows®. One-way analysis of variance (ANOVA) was used to compare the means of the multiple groups. Tukey HSD post hoc analysis was used for in-between group comparisons. a *P*-value of less than 0.05 is considered to be significant.

## Results

### Biochemical measurements in sera

#### Effect on serum CPK, CK-MB, and LDH levels

Celecoxib administration (50 mg/kg/day, orally), for 30 successive days was claimed to cause cardiotoxicity in male albino rats as manifested by significant elevation (*P* < 0.05) of serum CPK, CK-MB, and LDH levels, with ameliorative effects of royal jelly against celecoxib-induced cardiotoxicity (Table 1).

**Table 1:** Effects of celecoxib administration (50 mg/kg/day, orally), and celecoxib plus royal jelly administration (300 mg/kg/day, orally) in comparison to the control group, on cardiac profile in male albino rats. Data are expressed as mean ± SD, *n* = 10/group

Groups	Cardiac profile		
	CPK (U/l)	CK-MB (U/l)	LDH (IU/l)
1	271.33 ± 27.61^**c**^	278.00 ± 13.11^**c**^	179.67 ± 9.29^**c**^
2	943.00 ± 63.17^**a**^	816.00 ± 117.04^**a**^	470.33 ± 49.17^**a**^
3	503.33 ± 175.02^**b**^	612.83 ± 49.49^**b**^	326.00 ± 51.09^**b**^

Group (1) received no drug (control). Group (2) received celecoxib (50 mg/kg/day, orally), for 30 successive days. Group (3) received celecoxib (50 mg/kg/day, orally) and royal jelly (300 mg/kg/day, orally) for 30 successive days. Means carrying different superscripts in the same column are significant at *p* < 0.05

#### Effect on serum MDA and SOD levels

Celecoxib administration (50 mg/kg/day, orally), for 30 successive days was claimed to cause oxidative stress in male albino rats as manifested by significant elevation of serum MDA and significant decline in SOD levels, with ameliorative effects of royal jelly against celecoxib-induced oxidative stress (Table 2).

**Table 2:** Effects of celecoxib administration (50 mg/kg/day, orally), and celecoxib plus royal jelly administration (300 mg/kg/day, orally) in comparison to the control group, on serum MDA and SOD in male albino rats. Data are expressed as mean ± SD, *n* = 10/group

Groups	MDA (nmol/mL)	SOD (U/mL)
1	5.86 ± 0.66^**c**^	5.40 ± 0.62^**a**^
2	28.10 ± 3.48^**a**^	0.89 ± 0.68^**c**^
3	16.03 ± 2.51^**b**^	3.20 ± 0.61^**b**^

Group (1) received no drug (control). Group (2) received celecoxib (50 mg/kg/day, orally), for 30 successive days. Group (3) received celecoxib (50 mg/kg/day, orally) and royal jelly (300 mg/kg/day, orally) for 30 successive days. Means carrying different superscripts in the same column are significant at *p* < 0.05

### Apoptotic and anti-apoptotic genes expression

Celecoxib administration (50 mg/kg/day, orally), for 30 successive days was claimed to induce an apoptotic effect in male albino rats as manifested by a significant elevation of the serum apoptotic gene (Bax) and a significant decline in serum anti-apoptotic gene (Bcl-2) in liver, kidney, and heart with ameliorative effects of royal jelly against the celecoxib-induced apoptotic effect (Table 3).

**Table 3:** Effects of celecoxib administration (50 mg/kg/day, orally), and celecoxib plus royal jelly administration (300 mg/kg/day, orally) in comparison to the control group, on apoptotic and anti-apoptotic genes expression in male albino rats. Data are expressed as mean ± SD, *n* = 10/group

Groups	Apoptotic gene (Bax/ Gapdh %)	Anti-apoptotic gene (Bcl-2/Gapdh %)
	Heart	Heart
1	1.00 ± 0.00^**c**^	1.00 ± 0.02^**a**^
2	6.94 ± 1.15^**a**^	0.20 ± 0.07^**c**^
3	2.40 ± 0.39^**bc**^	0.68 ± 0.00^**b**^

Group (1) received no drug (control). Group (2) received celecoxib (50 mg/kg/day, orally), for 30 successive days. Group (3) received celecoxib (50 mg/kg/day, orally) and royal jelly (300 mg/kg/day, orally) for 30 successive days. Means carrying different superscripts in the same column are significant at *p* < 0.05

## Discussion

For over a century, nonsteroidal anti-inflammatory drugs (NSAIDs) have been among the most widely used treatments worldwide [16]. The anti-inflammatory drug celecoxib, the only inhibitor of cyclooxygenase-2 (COX-2 inhibitor) still used in the markets, for the treatment of various forms of arthritis and the management of acute or chronic pain due to its favorable gastrointestinal toxicity profile [17].

In our study, celecoxib administration (50 mg/kg/day, orally), for 30 successive days was claimed to cause cardiotoxicity in male albino rats with ameliorative effects of royal jelly against celecoxib-induced cardiotoxicity.

In fact, safety concerns, due to the elevated risk of myocardial infarction, caused all members of selective cyclooxygenase-2 (COX-2) inhibitors, except for celecoxib, to be withdrawn from the markets in 2004 to 2005 by the United States Food and Drug Administration (FDA) [18]. Celecoxib is still available on the market, nevertheless, as the FDA has determined its advantages outweigh any possible risks. Celecoxib has a significantly lower cardiovascular risk profile and the majority of research points to a low risk. The majority of early clinical studies using celecoxib in arthritic patients did not seem to indicate a rise in cardiovascular risk [19–21]. However, these trials tended to be short-term studies to evaluate pain management and the related adverse gastrointestinal events, with a relatively small proportion of patients at high cardiovascular risk or even excluded such patients entirely [22]. In a prospective, randomized, double-blind, multicenter, longer-term, placebo-controlled trial assessing the efficacy of celecoxib for the prevention of adenomatous polyps, celecoxib use was proved to be associated with a dose-related increase in cardiovascular harm (up to 3.4-fold) in form of myocardial infarction, stroke, heart failure, and even death [22].

A well-known diagnostic enzyme marker of myocardial damage is serum CPK, CK-MB, and LDH. Elevation of such cardiac markers indicates a significantly positive correlation between ischemic myocardial injury and inflammatory cell infiltration [23]. Following the WHO criteria for diagnosis of acute myocardial infarction (AMI), multiple cardiac biomarkers were used to diagnose acute myocardial infarction. Among such biomarkers are CPK, the oldest but non-cardiac specific marker, CK-MB as the most sensitive and specific marker for diagnosis of AMI, and cardiac reperfusion, and LDH, which is non-cardiac specific but the collaborated assays give an indication of myocardial damage [24]. Insufficient oxygen or glucose damages myocardial cells, tears heart membranes, or makes them permeable, which causes enzyme leakage and bloodstream entry [25] which indicates cardiotoxicity [26]. Rats exhibited increased serum cardiac enzyme levels of CPK, CK-MB, troponin-T, and LDH in return for celecoxib administration [27, 28].

The biochemical celecoxib-induced cardiotoxicity causes are surrounded by uncertainty. A widely accepted theory focuses on how celecoxib affects two important prostanoids, thromboxane A_2_ (TXA_2_) and prostacyclin (PGI_2_) which are essential for maintaining vascular homeostasis [29]. Thromboxane A_2_ is largely produced in platelets, which solely express COX-1 and promote platelet aggregation, vasoconstriction, and smooth-muscle proliferation. Prostacyclin, on the other hand, is the primary prostanoid product of endothelial cells and is produced as a result of COX-2 action. It has antiaggregative, antiproliferative, and vasodilatory properties [30]. Celecoxib may therefore suppress the vascular production of prostacyclin without affecting the synthesis of platelet-derived thromboxane A_2_. The imbalance between those two prostanoids leads to hypertension and atherosclerosis in a platelet-dependent mechanism creating a prothrombotic state [27]. Adenylyl cyclase (AC) enzyme is also inhibited by celecoxib, which leads to low levels of cyclic adenosine monophosphate (cAMP) [31]. The expression of thrombomodulin, a crucial inhibitor of blood coagulation, is downregulated in other platelet non-dependent pathways, which results in thrombosis [32]. Alternately, COX-2 suppression may cause a buildup of its substrate arachidonic acid, which results in the generation of harmful reactive oxygen species in the heart’s mitochondria [33]. Additionally, celecoxib eliminates the tissue factor (TF) expression attenuation caused by COX-2. The extrinsic coagulation pathway is vital for blood coagulation and thrombin production and is initiated by TF. As a result, TF is linked to thrombosis and cardiovascular system malfunction [34]. Respiratory chain enzyme (Complex IV) activity was significantly reduced following exposure to celecoxib. Parallel to this decline in complex IV activity, mitochondrial membrane potential (MMP) collapse, reactive oxygen species (ROS) production, mitochondrial swelling, ATP depletion, and lipid peroxidation occur [35].

In accordance with our findings, previous studies have demonstrated that elevated cardiac enzymes are usually reversible and will regain normal levels with time. In humans, CK-MB is detected in the serum 4 h after myocardial injury, peaks by 24 h, and normalizes within 48 to 72 h after myocardial ischemia, whereas the elevated CK levels may be detected for up to 60 h and then regain their baseline levels [36]. In rats, serum levels of CK-MB and LDH began to rise on day 1, reached their peak levels on day 7 after MI, and then declined gradually and normalized from day 21 onwards [37]. CK has a relatively short half-life (about 2–4 h), and thus activity rapidly returns to normal following cessation of myodegeneration or necrosis within a week [38].

The cardioprotective effect of royal jelly may be attributed to its efficient antioxidant and free radical scavenging capacity [39, 40]. Royal jelly (50, 100, and 150 mg/kg/orally, for 28 consecutive days) could remarkably reduce the paclitaxel-induced cardio-toxicity in adult male Wistar rats. It significantly reduced the cardiac biomarker creatine kinase (CK-BM) level and histopathological injuries such as diffused edema, hemorrhage, congestion, hyaline exudates, and necrosis. It also regained its total antioxidant capacity [10]. Royal jelly (250 mg/kg/orally, for 15 consecutive days) could significantly ameliorate the gamma radiation-induced cardio-toxicity in male albino rats. It remarkably regained normal serum-reduced glutathione (GSH) content, superoxide dismutase (SOD) activity, lipid peroxide [malondialdehyde (MDA)] levels, creatinine kinase-MB (CK-MB) levels, and cardiac troponin I level (cTnI). A somewhat normal appearance of cardiac muscle fibers was obtained thanks to RJ administration to radiation-exposed rats [11].

In our study, celecoxib administration (50 mg/kg/day, orally), for 30 successive days was claimed to cause oxidative stress in male albino rats as manifested by significant elevation of serum MDA and significant decline in SOD levels, with ameliorative effects of royal jelly against celecoxib-induced oxidative stress.

Oxidative stress (OS) is a shift in the balance between the production rate of oxidants and their elimination via the antioxidant defense system [41]. OS-mediated molecules are an array of metabolites derived from molecular reactive oxygen species (ROS) and reactive nitrogen species (RNS) [42]. Prime sites of ROS production in living organisms are the mitochondria, paroxysms, and endoplasmic reticulum. ROS are produced during the mitochondrial electron transport chain, through the oxidative phosphorylation pathway for ATP production [43]. ROS are also produced in phagocytes, auto-oxidation reactions, and subsequent to antioxidant enzymes (e.g., xanthine oxidase) [44]. RNS is derived from nitric oxide (^•^NO) metabolism [45]. ROS/RNS are produced from either endogenous or exogenous sources. Immune system activation, inflammation, mental stress, excessive exercise, cancer, infection, ischemia, and aging represent the endogenous stressors, while air pollution, water pollution, alcohol, cigarette smoke, heavy metals, pharmaceutical agents, and ultraviolet radiation act as exogenous sources of OS [46].

Antioxidants are the primary line of the body’s defense system against oxidative stress. In physiological conditions, antioxidants inhibit the oxidation reaction of molecules that can produce free radicals [47]. Several molecules play a role in such defense, either internally synthesized (endogenous), or externally supplied through foods (exogenous) antioxidants. Exogenous antioxidants, (Royal Jelly for instance), act as direct ROS scavengers and increase the antioxidant enzyme activities [48]. Endogenous antioxidant defenses involve a network of antioxidant enzymatic and non-enzymatic molecules that are usually disseminated within the cytoplasm and various cell organelles [49]. Primary antioxidant enzymes, such as superoxide dismutase (SOD), catalase, and several peroxidases catalyze a complex cascade of reactions to transform ROS into more stable molecules, such as water and O_2_. Malondialdehyde (MDA) is the end-product of polyunsaturated fatty acids oxidation in cellular membranes, thus it acts as a dependable marker of oxidative stress [50]. Non-enzymatic antioxidants include vitamins (A, C, E, and K), enzyme cofactors (Q10), and minerals (Zn, Mn, Cu, Se, etc.) [51].

In accordance with our findings, previous studies indicated an alteration in the oxidant/antioxidant status of rats treated with celecoxib [52–55]. The excessive generation of free radicals is considered one of the mechanisms by which celecoxib induces toxicity. Free radicals trigger the development of many diseases and cause harmful effects that cause the peroxidation of biomembranes and DNA, which is the reason for tissue destruction [56].

Several previous studies confirmed the concept of the beneficial and antioxidant effects of RJ [57–59]. Administration of royal jelly in vitro inhibited the production of pro-inflammatory cytokines such as TNF-α and IL-1, IL-6 in a dose-dependent manner [60]. Other in-vivo and in-vitro studies also proposed anti-inflammatory and anti-oxidant properties of royal jelly [61]. The Antioxidant activity of RJ can be attributed to the free radical scavengers; they can inhibit lipid peroxidation [62] via scavenging hydroxyl radicals [63], and they attributed this activity to three dipeptides containing tyrosine residues. Additionally, RJ inhibits the enzymes that elevate the peroxidation of endogenous lipids, as well as cytochrome P450 expression, which is one of the cellular sources of oxygen radicals [64].

In our study, celecoxib administration (50 mg/kg/day, orally), for 30 successive days was claimed to induce an apoptotic effect in male albino rats as manifested by a significant elevation of serum apoptotic gene (Bax) and a significant decline in serum anti-apoptotic gene (Bcl-2) in liver, kidney, and heart, with ameliorative effects of royal jelly against the celecoxib-induced apoptotic effect.

Apoptosis is programmed cell death, which involves the genetically determined elimination of cells. Since too little or too much cell death can result in pathology, such as autoimmune illnesses, neurodegeneration, or cancer, it is obvious that apoptosis needs to be strictly gene-regulated [65]. Several factors contributed to the apoptotic mechanism, among them, two main families of proteins including caspases and the Bcl-2 family. Caspases, death-driving cysteine proteases, play an effective role in the apoptotic process. Activation of caspases ensures that the cellular components are degraded in a controlled manner, carrying out cell death with minimal effect on surrounding tissues [66]. Whereas, Bcl-2, consists of anti-apoptotic and pro-apoptotic members. The anti-apoptotic members of this family, such as Bcl-2 and Bcl-XL, prevent apoptosis either by sequestering caspases or by preventing the release of mitochondrial apoptogenic factors such as cytochrome c and apoptosis-inducing factor (AIF) into the cytoplasm, which are responsible for caspases activation. In contrast, pro-apoptotic members of this family, such as Bax and Bak, trigger the release of caspases by inducing the release of mitochondrial apoptogenic factors into the cytoplasm leading to caspases activation [67].

Apoptosis is an essential physiological process for the selective elimination of individual cells without destruction or damage to the whole organ [68]. The morphological features of apoptosis include cell shrinkage and pyknosis, cytoplasmic and nuclear condensation, chromatin cleavage, apoptotic bodies formation, an intact plasma membrane, and exposure of surface molecules to phagocytosis [69]. In contrast, necrosis has been characterized as passive, accidental cell death that is triggered by external factors or disease with the uncontrolled release of inflammatory cells [70, 71]. The main morphological changes associated with necrosis include cell swelling, cytoplasmic vacuole formation, distended endoplasmic reticulum, cytoplasmic bleb formation, condensed, swollen, or ruptured mitochondria, disrupted organelle membranes, swollen and ruptured lysosomes, and ultimately disruption of the cell membrane [72]. While necrosis is always pathological and detrimental to an organ [65], apoptosis is a normal and physiological process to get rid of certain cells that have been damaged beyond repair. is almost always normal and advantageous. Additionally, apoptosis happens as a protective process, such as in immunological responses or when diseases or toxic chemicals destroy cells. Thus, it keeps normal tissue function [73].

In accordance with our findings, previous studies have confirmed celecoxib-induced apoptosis. Celecoxib causes apoptosis by causing the loss of the mitochondrial transmembrane potential, the release of cytochrome c and AIF, and the activation of caspase-9 and caspase-3. Additionally, the anti-apoptotic protein Bcl-2 was reduced in abundance whereas the pro-apoptotic protein Bax was enhanced by celecoxib. The data showed that mitochondria-dependent signaling, not PPAR/NF-B signaling, was the mechanism through which celecoxib triggered apoptosis in mouse liver cancer cells [74]. In another study, celecoxib induced apoptosis in 5-fluorouracil-resistant gastric cancer cells through protein kinase B (PKB) inhibition [75], which is a key component of the phosphatidyl-inositol-3 kinase (PI3K) intracellular pathway that exerts a pivotal role in regulating cell proliferation, survival, and metabolism [76]. Celecoxib induced apoptosis in glioblastoma tumor cells, the primary malignant tumor of the brain, via suppressing CIP2A/PP2A/Akt signaling axis [77].

Several previous studies confirmed the concept of the beneficial and anti-apoptotic effect of RJ against cisplatin-induced hepatorenal toxicity [78], nicotine-induced testicular injury in mice [79], doxorubicin-induced nephrotoxicity in male albino rats [80], and hydroxyurea-induced hepatic injury in rats [81]. Moreover, Royal jelly decreases the expression of the apoptotic gene (MMP-9) responsible for bladder cancer in humans [82].

More generally, this study highlights the necessity of better techniques for evaluating potential adverse cardiovascular outcomes of celecoxib, as well as the necessity of long-term, placebo-controlled trials to evaluate safety in addition to efficacy of royal jelly as a concomitant drug.

## Conclusion

The study found that long-term administration of celecoxib caused cardiovascular complications in male albino rats. Royal jelly was found to significantly decrease serum levels of creatine phosphokinase (CPK), creatine kinase-MB (CK-MB), and lactate dehydrogenase (LDH). It also showed a significant decrease in the oxidative stress indicator malondialdehyde (MDA) levels and the bax gene. Additionally, it demonstrated significant increases in the bcl-2 gene and superoxide dismutase (SOD) levels, which contribute to its therapeutic effects against celecoxib-induced cardiotoxicity.

## Data Availability

All relevant data are included in this published article.

## Declarations

### It is declared that

All methods were carried out in accordance with relevant guidelines and regulations. It was performed according to the recommendations of Good Clinical Practice and the Declaration of Helsinki (2013).
